# Crime and violence in Brazil: Systematic review of time trends, prevalence rates and risk factors^☆^

**DOI:** 10.1016/j.avb.2013.07.003

**Published:** 2013-09

**Authors:** Joseph Murray, Daniel Ricardo de Castro Cerqueira, Tulio Kahn

**Affiliations:** a Department of Psychiatry, University of Cambridge, Douglas House, 18b Trumpington Road, Cambridge CB2 8AH, United Kingdom; b Instituto de Pesquisa Econômica Aplicada, Brazil; c Fundação de Estudos e Formação Política do Partido Social Democratico, Brazil

**Keywords:** Crime, Violence, Systematic review, Prevalence, Risk factors, Middle-income country

## Abstract

Between 1980 and 2010 there were 1 million homicides in Brazil. Dramatic increases in homicide rates followed rises in inequality, more young men in the population, greater availability of firearms, and increased drug use. Nevertheless, disarmament legislation may have helped reduce homicide rates in recent years. Despite its very high rate of lethal violence, Brazil appears to have similar levels of general criminal victimization as several other Latin American and North American countries. Brazil has lower rates of drug use compared to other countries such as the United States, but the prevalence of youth drug use in Brazil has increased substantially in recent years. Since 1990, the growth of the Brazilian prison population has been enormous, resulting in the fourth largest prison population in the world. Through a systematic review of the literature, we identified 10 studies assessing the prevalence of self-reported offending in Brazil and 9 studies examining risk factors. Levels of self-reported offending seem quite high among school students in Brazil. Individual and family-level risk factors identified in Brazil are very similar to those found in high-income countries.

## Highlights

• Between 1980–2010 the Brazilian homicide rate rose to one of the highest worldwide.
• Levels of non-lethal victimization in Brazil seem similar to other American nations.
• Rates of self-reported offending are quite high among school children in Brazil.
• Individual and family risk factors are similar to those in high-income countries.
• More systematic official and self-report crime data collection is needed in Brazil.

## 1 Introduction

In 2008, 535,000 people died by homicide worldwide and 95% of these deaths occurred in low and middle-income countries (LMIC) (World Health Organization, 2012). Leading criminologists have argued for more globalized research on crime and violence (Farrington, 2000; Laub & Lauritsen, 1993). However, research programs continue to focus almost exclusively on high-income countries. In a review of the most important longitudinal studies in criminology (Farrington & Welsh, 2007), only two were identified in LMIC: in Mauritius (Raine, Reynolds, Venables, Mednick, & Farrington, 1998) and China (Taylor, Friday, Ren, Weitekamp, & Kerner, 2004). The rest were conducted in North America (20 studies), Northern Europe (11 studies), and Australasia (4 studies). Basic information is often hard to obtain on the prevalence and correlates of crime in LMIC. In this article, we review epidemiological evidence on crime and violence in Brazil, a middle-income country with an extremely high homicide rate.

Brazil has the fifth largest population in the world: 197 million people (World Bank, 2012b), 30% of whom are under age 18 (Unicef, 2012). Although Brazil's gross national income is not low (US$ 11,500 per capita in 2011, World Bank, 2012a), it has persistently had one of the highest rates of inequality in the world: its 2012 GINI index (51.9) was the 16th highest out of 136 countries worldwide (the United States ranks 42nd, and the United Kingdom ranks 91st) (Central Intelligence Agency, 2013). In 2009, the poorest fifth of the population received just 2.9% of the nation's income compared to 58.6% received by the richest fifth (World Bank, 2012c). In the same year, 10.9% of the nation's population was poor (living on less than $2 per day; World Bank, 2012c). Nevertheless, there have been considerable improvements in health outcomes in Brazil in recent decades: between 1975 and 2007 infant mortality decreased from 114 to 19 (per 1000 live births), and life expectancy increased from 52 to 73 years between 1975 and 2008 (Paim, Travassos, Almeida, Bahia, & Macinko, 2011). Access to education also increased substantially: the proportion of people with seven or more years of formal education increased from 19% to 47% between 1976 and 2008 (Paim et al., 2011). However, ranking 85th out of 187 countries on the Human Development Index in 2011 (this index combines indicators of life expectancy, educational attainment and national income; United Nations, 2012), Brazil still has considerable challenges in meeting the whole population's needs for education, health care, and income.

Violence has grown into a major public health problem in Brazil. From the 1930s, infectious diseases accounted for an increasingly smaller proportion of deaths, while deaths caused by violence steadily increased (Barreto et al., 2011). In 2007, 12.5% of all deaths were caused by violence, mostly among young men (Reichenheim et al., 2011). Between 1997 and 2007, the prison population in Brazil grew faster than any other American country (Walmsley, 2010). By 2011, a total of 515,000 individuals were incarcerated in Brazil (270 per 100,000 population, Ministerio de Justiça, 2012), corresponding to the fourth largest prison population in the world after the United States, China, and Russia (International Centre for Prison Studies, 2012).

The social and economic costs of crime and violence in Brazil are large. Injuries (Gawryszewski & Rodrigues, 2006), fear (Cardia, 2012), and psychological health problems (Andrade et al., 2012; Lopes, Faerstein, Chor, & Werneck, 2008) have profound impacts on individuals' quality of life. Wider societal costs, including expenditure on healthcare and public and private security, can be expressed as a percentage of gross domestic product (GDP). Summing expenditure on police, prisons, private security, public health, and loss of human capital (from premature deaths caused by violence), and personal loss from robbery and theft, the total cost of crime in Brazil was estimated to be R$92 billion in 2004, or 5.1% of GDP (Cerqueira, Carvalho, Lobão, & Rodrigues, 2007). Human capital costs of homicide alone were equal to 2.3% of GDP in 2007 (Cerqueira, 2010).

According to the landmark World Report on Violence and Health (World Health Organization, 2002) the first steps needed towards violence prevention are: (i) collection of as much basic knowledge as possible on the magnitude and nature of the problem, and (ii) research identifying causes and correlates of violence which might be modified through interventions. Given its economic, cultural, and social context, it is possible that risk factors for crime identified in other contexts have different effects in Brazil, and this needs empirical testing (Rutter, 1999). For example, Villarreal and Silva (2006) found a *positive* association between levels of neighborhood social cohesion and crime rates in urban Brazil, although the relationship is generally *negative* in the United States. The positive association in Brazil might arise because the survival of poor urban settlements depend on inhabitants organizing to prevent governments removing them, as well as inhabitants having shared histories of migration from the countryside, and depending on each other to survive in the informal sector — to secure jobs and build homes (Villarreal & Silva, 2006). Therefore, although impoverished neighborhoods in Brazil do have high crime rates, they tend to have unusually high levels of social cohesion, unlike in North America. As this example shows, it cannot be taken for granted that correlates of crime identified in high-income countries will replicate in Brazil.

Homicide statistics are probably the most reliable data on violence in Brazil, and are a key resource for public debate about violence prevention. Although there is a general correlation between violence levels (including homicide) and other types of crime (Eisner, 2002), it is possible for nations with high homicide rates to have average or low crime rates and vice versa (Savolainen, Lehti, & Kivivuori, 2008; Zimring & Hawkins, 1999). Therefore, as well as examining rates and trends in homicide in Brazil, we systematically review evidence on non-lethal crime and violence. Given that discussions of macro-level correlates of crime in Brazil are available elsewhere (e.g., Cerqueira, 2010; Villarreal & Silva, 2006), we focus our review on risk factors for criminal behavior measured at the individual level. For a helpful general discussion of criminology in Brazil, see Rodrigues (2011).

Much of what we review is difficult to access by international researchers given its publication in Portuguese in national reports or journals. This issue of language may partly explain criminology's lack of focus on countries like Brazil, despite their high levels of serious violence. For example, in the enormous Handbook of Crime Correlates (Ellis, Beaver, & Wright, 2009) only three Brazilian studies are cited (two of which pertain to alcoholism rather than crime). While this is a useful start, a comprehensive review of crime and violence in Brazil requires systematic searches of local as well as international sources, in Portuguese as well as in English. We hope that the current systematic review will help bring the problems of crime and violence in countries like Brazil closer to the hub of international criminological research.

### 1.1 Objectives of the review

The review has the following five objectives:

1. Compare homicide rates and the overall burden of violence in Brazil with other countries worldwide.
2. Examine time trends in homicide in Brazil.
3. Examine rates of non-lethal criminal victimization in Brazil over time and in comparison with other countries.
4. Systematically review community based studies on the prevalence of offenders in Brazil.
5. Systematically review community based studies of risk factors for crime in Brazil.

## 2 Methods: primary data sources

### 2.1 Homicides in WHO member states

Homicide rates (per 100,000 people) for member states of the World Health Organization (WHO) in 2008 were extracted from the Global Burden Disease 2004 Update (World Health Organization, 2008).^1^ Homicide rates for member states are based on vital registration data classified using the codes X85 to Y09 and Y87.1 in the International Classification of Diseases 10th Edition (ICD-10).

### 2.2 Disability-Adjusted Life Years in WHO member states

We use Disability-Adjusted Life Years (DALY) to measure the total burden of life years lost to violence in Brazil and other WHO member states. One DALY can be thought of as one year of “healthy” life lost because of premature mortality or morbidity (injury or ill health) caused by violence. DALYs caused by violence for WHO member states in 2004 (the most recent year available) are examined in this article. As described by the WHO, DALYs “are calculated as the sum of the years of life lost due to premature mortality (YLL) in the population and the years lost due to disability (YLD) for incident cases of the disease or injury” (for further details, see World Health Organization, 2008, p. 3).

### 2.3 National population of Brazil

National population estimates for 1980–2010 (used to express crime rates in terms of 100,000 people) were taken from the Brazilian Department of Public Health Information (DATASUS). Population counts between 1980 and 2010 are based on censuses carried out by the Brazilian Institute of Geography and Statistics (IBGE) in 1980, 1991, 1996, 2000, and 2010, with intervening years imputed using age and sex stratifications.

### 2.4 Homicides in Brazil

We use data on homicides from the Brazilian System of Death Registration (SIM) maintained by the Brazilian Ministry of Health (Ministério da Saúde). Data were extracted from the Department of Public Health Information (DATASUS). This is widely regarded as the most reliable information source on homicides in Brazil, even though unregistered deaths and deaths with unknown causes are still problematic (Cerqueira, 2012; de Souza, de Lima, & Bezerra, 2010). For the years 1979–1995, all deaths with codes E960–E969 in the International Classification of Diseases 9th Edition (ICD-9) were counted as homicides. For the years 1996–2010, all deaths with codes X85 to Y09 and Y87.1 in ICD-10 were counted as homicides. This corresponds with the coding of violent deaths in the Global Burden Disease 2004 Update (World Health Organization, 2008). We also report estimated homicide rates that account for deaths by external causes that have unknown intent. These estimates are based on models of the probability that violent deaths with unknown intent (which are high in some Brazilian states) are actually homicides, taking into account recorded individual characteristics and situational aspects of the death (see Cerqueira, 2012, for methodological details).

### 2.5 Non-lethal hospitalizations caused by violence in Brazil

The Brazilian System of Information on Public Hospitals (SIH-SUS) maintains data on admissions to public hospitals in Brazil, with causes of admission coded using ICD-9 until 1996 and using ICD-10 afterwards. We examine rates of public hospital admissions caused by violence not resulting in death in hospital per 100,000 people. Admissions caused by violence were identified as ICD-9 codes E960–E969 and ICD-10 codes X85 to Y09. Ideally, data on violence-related treatment in hospital emergency departments would also be used to examine rates of violence in Brazil. However, Brazilian emergency department information systems are currently inadequate to provide comprehensive national data (Gawryszewski et al., 2008).

### 2.6 Criminal justice data in Brazil

State police force data on numbers of crimes and offenders are compiled to produce national statistics by the Brazilian Ministry of Justice (Secretaria Nacional de Segurança Pública, Ministério da Justiça). However, many states provide very incomplete data. The Brazilian Forum on Public Security (Fórum Brasileiro de Segurança Pública, 2011) classifies each state as having low, medium, or high quality data, according to the extent of its data coverage and the proportion of deaths with undetermined causes. We examine statistics both for all states and separately for those states classified as having high quality data. Data on the number of offenders recorded by the police in Brazil in 2009 were provided by the Brazilian Ministry of Justice (Secretaria Nacional de Segurança Pública, Ministério da Justiça, personal communication, 2011).

The Prison Department of the Brazilian Ministry of Justice (Informações do Departamento Penitenciário Nacional, Ministério da Justiça) collects state prison data to produce national statistics on the number of people incarcerated in adult prisons, crimes committed by the incarcerated population, and basic demographics of the population.

## 3 Methods: systematic review of literature

We systematically searched for community based studies in Brazil reporting the prevalence of criminal offending, or associations between risk factors and offending. We report on a similar synthesis of evidence about childhood conduct problems in Brazil in Murray, Anselmi, Gallo, Fleitlich-Bilyk, and Bordin (2013). To be eligible for the current review, the study must have met all four of the following criteria:

1. The study used a community based sample with random, stratified probability, or total sampling in households, schools, or maternity hospitals (in the case of birth cohort studies). Studies that recruited samples entirely in an institutionalized setting, for example a drug addiction center, were excluded.
2. The study had at least 100 participants.
3. The study measured criminal behavior at the individual level using self-reports, other reports, or criminal records. Studies of domestic violence and suicide were not included.
4. The study reported either the prevalence of crime or the association between at least one risk factor and crime.

**Fig. 1:**
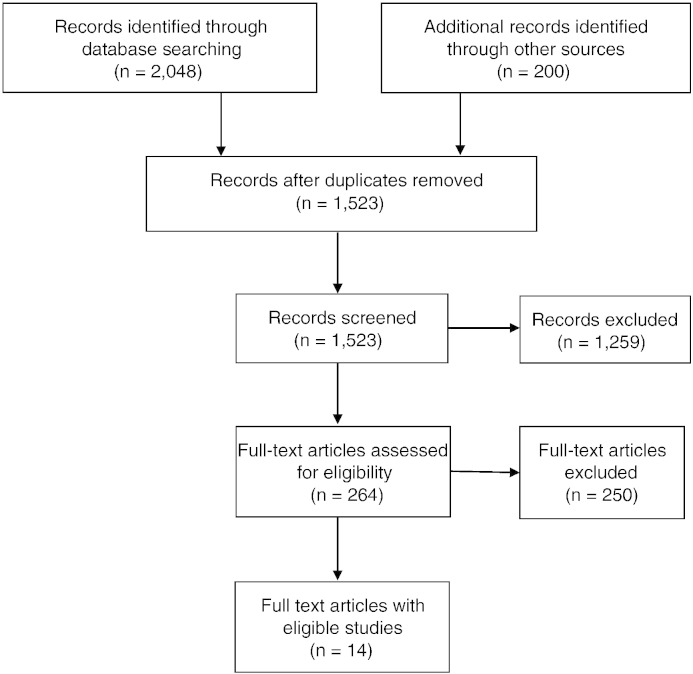
Flowchart of screening process to identify eligible studies for the review.

Studies could be reported in English or on Portuguese. Published and unpublished studies were eligible. We searched the following electronic databases for eligible studies in May 2012: Social Science Citation Index, PubMed, and Lilacs. Lilacs is the largest database of scientific literature in Latin America and the Caribbean. The following keywords were used (and they were also translated and entered into Lilacs separately in Portuguese): [ODD OR Oppositional Defiant Disorder OR CD OR Conduct disorder OR Conduct problems OR Externalizing OR Crime OR Violence OR Delinquency OR Illicit Drug* OR Substance use OR Substance abuse] AND [Cohort OR Longitudinal OR Prospective OR Cross sectional OR Case control OR Population] AND [Prevalence OR Rate OR Incidence OR Frequency OR Risk factor] AND Brazil. To the list of references retrieved from electronic searches, we also added documents from our own archives, internet searches, recommendations from Brazilian researchers, and articles in references lists of retrieved reports. A flow chart of the search and screening process is shown in Fig. 1.

Two researchers independently assessed the full texts for eligibility. On first assessment there was 88% agreement on the studies that should be included and excluded; remaining studies were re-examined and discussed to agree on inclusion or exclusion.

## 4 Results

### 4.1 Homicide trends and cross-national comparisons

LMIC in Africa and the Americas have the highest homicide rates in the world and bear the greatest burden of violence in terms of Disability Adjusted Life Years (see Table 1). In 2008, the average homicide rate worldwide was 7.9 per 100,000 people. The rate of 29.6 in Brazil was the 13th highest rate out of all 193 WHO member states. Between 1980 and 2010, there were a total of 1.09 million homicides in Brazil. In 2004, an estimated 2.5 million healthy life years were lost due to violence (2,488,000 DALYs) in Brazil. This was the highest burden of life years lost to violence in any WHO member state in that year.

**Fig. 2:**
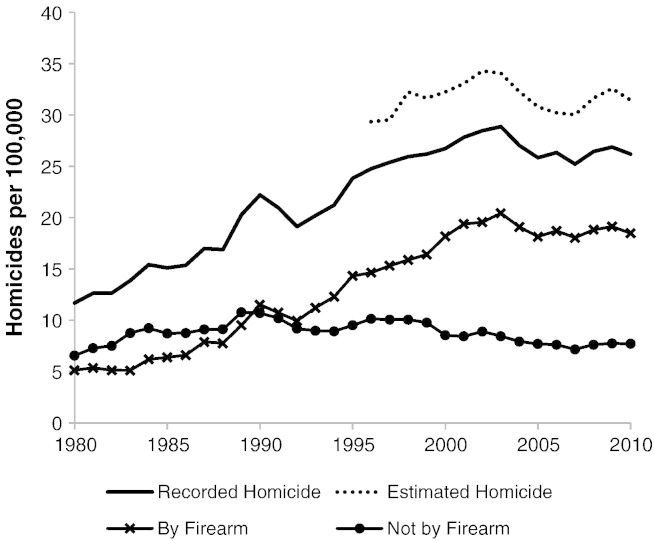
Homicide trends in Brazil 1980–2010.

Source: Brazilian System of Death Registration (SIM), Department of Public Health Information (DATASUS). Estimated homicide rates modeled by Daniel Cerqueira (see Cerqueira, 2012, for details of methodology).

Fig. 2 shows the enormous escalation in recorded homicide rates in Brazil over the last three decades. Homicides per 100,000 increased from 11.7 in 1980 to a peak of 28.9 in 2003, followed by a slight decline thereafter to 26.2 in 2010. There were 50,000 homicides in Brazil in 2010. The increase in homicide rates has occurred almost entirely among young men (Waiselfisz, 2010). Between 1980 and 2010, the rate of youth (under 20 years old) homicide increased by 346% (Waiselfisz, 2012).

Modeled data, accounting for deaths by external causes with unknown intent, show that actual homicide rates in Brazil may be significantly higher than recorded figures (see years 1996–2010 in Fig. 2). The estimated homicide rate in 2010 was 31.5 homicides per 100,000 people (compared to the recorded rate of 26.2).

Fig. 2 also shows that changes in the total homicide rate followed in lock step with changes in the rate of homicide caused by firearms. Notably, after a steady increase in homicide rates during the 1990s and early 2000s there was a downturn from 2004 onwards, after disarmament legislation in 2003. In contrast, the rate of homicide not involving firearms remained relatively stable from the mid-1980s onwards.

Two studies evaluated longer-term trends in deaths by violence in Brazil. Vermelho and Jorge (1996) examined causes of death among 15–24 year olds in Rio de Janeiro and São Paulo between 1930 and 1991. From the 1930s youth mortality rates fell as infectious diseases were controlled (infectious diseases had been the leading cause of death). However, from the 1960s, youth mortality rates actually increased again, with violence becoming the leading cause of death among 15–24 year olds. Barreto et al. (2011) also showed that the proportion of all deaths caused by violence in the whole of Brazil grew steadily from 1930 to 2007, while the proportion of deaths caused by infectious diseases decreased dramatically.

In Brazil, homicide victims are most likely to be young, male, Black, and with few years of education, as shown in the following statistics. In 2009, the male homicide rate (51.1 per 100,000) was over 10 times the female rate (4.3 per 100,000). The homicide rate was highest for black people (34.6), then indigenous (32.5), white (16.3) and people of Asian descent (6.8). By age group, rates were: 24.0 (10–19 years); 62.5 (20–29 years); 40.3 (30–39 years); 25.5 (40–49 years); 14.3 (50–59 years); and 9.2 (60 + years). Homicide rates by number of school years completed were: 30.3 (1–3 years) 36.1 (4–7 years), 13.1 (8–11 years), and 7.8 (12 + years).

A series of careful examinations of the geography of Brazilian homicides shows that there are large regional variations in rates and trends (Waiselfisz, 2007, 2008, 2010). Until 1999, the highest rates of homicide were concentrated in state capitals and metropolitan regions; however, thereafter, rates stabilized in those areas (mainly in São Paulo) in contrast to a persistent upturn in interior and countryside regions.

In addition to homicides by civilians, there were 1829 persons killed by military or civil police in 17 states with available data^2^ in 2010 (Fórum Brasileiro de Segurança Pública, 2011). This is equivalent to a rate of 1.6 persons killed by the police per 100,000 people in those states, higher than the homicide rate in England and Wales (1.2 per 100,000 in 2010–11, Smith, Osborne, Lau & Britton, 2012).

### 4.2 Non-lethal criminal victimization

#### 4.2.1 Non-lethal violence recorded in the health system

**Fig. 3:**
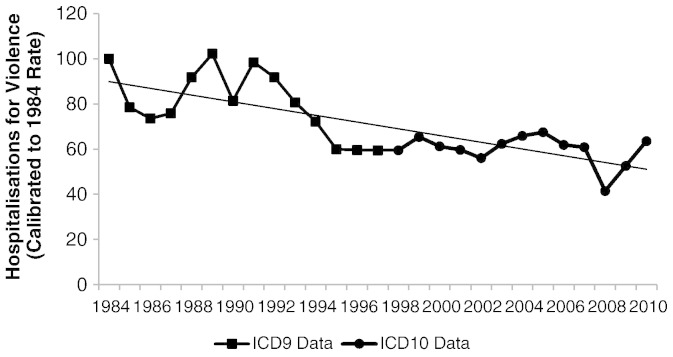
Trends in hospitalizations for non-lethal violence 1984–2010.

Source: Brazilian Ministry of Health, System of Information on Public Hospitals (SIH-SUS). Straight line shows linear trend in hospitalization rates.

Fig. 3 shows an overall decline in rates of admission to public hospitals for assault (not resulting in death) between 1984 and 2010.^3^ Although one must consider that admissions to private hospitals are not included (equal to 30% of all hospital admissions in Brazil in 2008), the downward trend in public hospital admissions for violence since the late 1990s is not explained by increases in private hospital admissions. In fact, the proportion of all people admitted to hospital who were admitted to private hospitals was 39% in 1998, 37% in 2003 and 30% in 2008.^4^

Gawryszewski and Rodrigues (2006, p. 210) found that men were five times more likely to be hospitalized because of assault than women and the highest rate of admission for assault was among 15–34 year olds, in 2003. Assaults were the only type of injury cause for which the number of fatalities was greater than the number of nonfatal victims, which Gawryszewski and Rodrigues suggested was because of the high proportion of firearms used in assaults (71% in all homicides and 30% in non-fatal assaults).

Two studies examined injuries caused by violence in a survey of 65 hospital emergency departments between September and November 2006 (Gawryszewski et al., 2008; Mascarenhas et al., 2009). Among victims of aggression, 31% were admitted to hospital (67% were treated and released and 1% died). Thirty-six percent of victims had been injured by a stranger. An overwhelming majority of aggressors (72%) were identified as male (9.4% female, 2.3% both male and female, and 16.3% without information).

#### 4.2.2 Police recorded crimes

In 2009, the rate of non-lethal crimes recorded by the police in Brazil was (per 100,000 people): 19 attempted homicides, 538 robberies, 340 assaults, 13 rapes, 29 crimes of drug possession, 38 crimes of drug trafficking, and 16 crimes of firearm possession.^5^ Comparing rates of non-lethal assault (340 per 100,000) and homicide (26.9 per 100,000) in 2009, it is clear that mortality represents only “the tip of the iceberg of Brazilian violence” (de Souza & de Lima, 2006, p. 365). It is also likely that police statistics greatly underestimate true levels of crime in Brazil. In the Brazilian National Household Survey 2009 (PNAD, Instituto Brasileiro de Geografia e Estatística, 2010b), under half of crime victims reported the incident to the police: 48% for robbery, 38% for theft, and 44% for assault. Note that these reporting rates are similar to those in other countries in the International Crime Victim Survey 2004–05, in which average reporting rates were: 46% for robbery, 46% for theft of personal property, 33% for assault and threat (Dijk, Kesteren, & Smit, 2007, p. 267).

#### 4.2.3 Victimization survey: Brazilian National Household Survey

The fact that many crime victims do not report crimes to the police emphasizes the importance of victimization surveys for assessing the prevalence of non-lethal crime. We review results from Brazilian victimization studies that used nationally representative samples, repeated surveys to examine time trends, or comparable data from other countries.

The largest surveys to have assessed criminal victimization in Brazil are the Brazilian National Household Surveys (PNAD, Instituto Brasileiro de Geografia e Estatística, 2010b). These surveys are conducted annually (except in census years) and cover a wide range of social and economic topics. In 1988 and 2009, several identical questions were included about crime victimization (referring to the previous 12 months), which we examine for respondents aged 10 years and above. In 2009, 7.4% of respondents reported being a victim of robbery or theft compared to 5.4% in 1988 (representing a 37% increase). In 2009, 1.6% reported being a victim of physical aggression compared to 1.0% in 1988 (60% increase). Rates of attempted robbery or theft were 5.4% in 2009 and 1.6% in 1988 (237% increase). Thus, on all three indicators of victimization there were substantial increases between 1988 and 2009.

In the 2009 PNAD survey, victims of robbery, theft, and physical aggression were more likely to be male than female, and were most likely to be young (16–24 years old) (Instituto Brasileiro de Geografia e Estatística, 2010b). Although being victim of robbery or theft was more likely among rich people (11.6% victimization rate for people with 5 + minimum wages; 4.7% for people with up to 1/4 minimum wage), physical aggression was more likely among poor people (1.0% for people with 5 + minimum wages; 2.2% for people with up to 1/4 minimum wage). Robbery and physical aggression were most likely to occur on the street, while theft was most likely to occur at home.

#### 4.2.4 Victimization survey: Núcleo de Estudos da Violência

The Núcleo de Estudos da Violência (Universidade de São Paulo) surveyed 1600 people (aged over 15 years) in 10 Brazilian state capitals in 1999, and 4205 people in the same cities (and also in Fortaleza) in 2010 (Cardia, 2012). Rates of 12-month victimization in 2010 and 1999 were, respectively: 4.2% and 5.8% for physical aggression; 0.6% and 0.7% injury by firearm; 7.2% and 5.8% threatened with a gun during a robbery; 2.6% and 4.0% threatened with a knife during a robbery; 7.7% and 7.5% offered drugs; 2.1% and 3.1% threatened by police to extract money.

#### 4.2.5 Victimization survey: AmericasBarometer

AmericasBarometer is one of the largest surveys of public opinion in the Americas, including national probability samples of adults in 26 countries. In 2010, 2482 voting-age adult Brazilians were included in the face-to-face survey. The core questionnaire included the following question on crime victimization “Have you been a victim of any type of crime in the past 12 months? That is, have you been a victim of robbery, burglary, assault, fraud, blackmail, extortion, violent threats or any other type of crime in the past 12 months?” Overall in the Americas, 19.3% of people responded positively to this question (Seligson & Smith, 2010). The highest rates of victimization were in Peru and Ecuador (31.1% and 29.1% respectively) and the lowest rates were in Guyana and Jamaica (9.0% and 10.1% respectively). Brazil had the 18th highest level of crime victimization (15.8%). Other countries with similar rates to Brazil were Canada (15.3%) the United States (16.4%), the Dominican Republic (16.5%) and Chile (16.7%). Brazil had the fifth highest rate of corruption (being asked to pay a bribe in the year prior to the survey). In Brazil, 23.6% of respondents said that they had been asked to pay a bribe, compared to 53.6% in Haiti (the country with the highest rate) and 4.2% in Canada (the country with the lowest rate).

#### 4.2.6 Victimization survey: Latinobarómetro

**Fig. 4:**
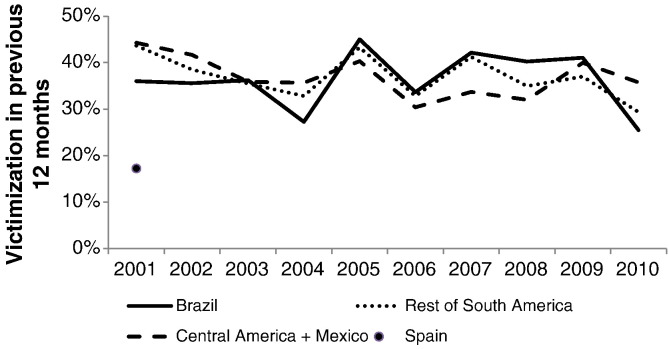
Trends in non-lethal criminal victimization in Brazil and Latin America: 2001–2010.

Source: Our analyses of Latinbarómetro data (Latinbarómetro, 2012).

Latinobarómetro is an annual public opinion survey using stratified samples of between 1000 and 1200 people in 18 different Latin American countries. In Brazil, sampling was designed to be representative of all persons aged 16 and over in surveys since 2001.^6^ Each year, the survey included the following question on crime victimization: “Have you, or someone in your family, been assaulted, attacked, or been the victim of a crime in the last 12 months?” According to this survey, victimization among households in Latin America in 2001 was much more common than in Spain: 36% in Brazil, 43% in the rest of South America, 44% in Central America and Mexico, and 17% in Spain (our calculations: Latinbarómetro, 2012). Trends in criminal victimization between 2001 and 2010 were similar in Brazil to those in the rest of South and Central America (see Fig. 4). Notwithstanding year-on-year variation, which may reflect sampling error, rates of overall household victimization appear relatively stable through this period.

#### 4.2.7 International Crime Victim Survey

The International Crime Victims Survey (ICVS) is a program of surveys about crime victimization using standardized questionnaires designed to maximize comparability between countries. Brazil has not conducted national crime victimization surveys as part of this program, but in 2002, the standardized questionnaire was applied to samples in the cities of São Paulo, Rio de Janeiro, Recife and Vitória. Probabilistic sampling was used to select 700 participants in each city. Kahn, Besen, and Custódio (2002) compared average rates of victimization in these four Brazilian cities with average rates in 17 European and North American countries in 2000. Victimization rates in the previous 12 months were higher in Brazil than in Europe and North America for the following types of crime: theft of car (6.2% versus 1.0%), theft of motorbike (9.8% versus 0.3%) theft of bicycle (8.1% versus 3.2%), theft from car (6.4% versus 4.6%), robbery (5.5% versus 0.8%), and attempted burglary (2.2% versus 1.8%) (Kahn et al., 2002, p. 7).^7^ Rates of victimization were lower in Brazil than in Europe and North America for the following crimes: theft of personal property (3.0% versus 3.9%), physical assault (2.5% versus 3.5%), burglary (1.5% versus 1.8%), and sexual crimes (1.4% versus 1.7%). Similar patterns of results are apparent comparing victimization rates in São Paulo and Rio de Janeiro (in the 2002 survey) with the main cities in the 2004–2005 International Crime Victims Survey (see Dijk et al., 2007, pp. 241–242).

### 4.3 Prevalence of criminal offenders in Brazil

In Sections 4.1 and 4.2, we reviewed data on the frequency of lethal and non-lethal criminal *victimization* in Brazil. In this section, we review evidence on the prevalence of criminal *offenders* in Brazil.

#### 4.3.1 Number of offenders recorded by the police

The total number of offenders recorded by the police in Brazil in 2009 was 714,185 (373 offenders per 100,000 people). However, seven states contributed no data to this national statistic, and the data coverage in many other states is believed to be poor. Considering only the nine states with data classified as high quality,^8^ 245,000 offenders were recorded in 2009 (989 offenders per 100,000 people).

#### 4.3.2 Number of incarcerated offenders

In the last two decades, the Brazilian adult prison population grew enormously. The number of incarcerated men (per 100,000 over 18 years old) was: 129.3 in 1989, 176.3 in 1995, 275.7 in 2000, and 466.4 in 2009 (Fórum Brasileiro de Segurança Pública, 2011). The number of incarcerated women (per 100,000 over 18 years old) was: 4.5 in 1989, 6.6 in 1995, 12.0 in 2000, and 33.2 in 2009. The number of juvenile offenders (12–17 year olds detained in young offenders' institutions) increased from 4245 in 1996 to 17,703 in 2010 (equivalent to 88 per 100,000 in 2010) (Secretaria de Direitos Humanos, 2011).

#### 4.3.3 Prevalence of self-reported offenders in community based studies

In a systematic review of the literature, we located 10 Brazilian studies that used self-report questionnaires to assess the prevalence of offenders in community based samples. These studies are summarized in Table 2. Seven were surveys of school children (7–19 years old), one was a household survey of adolescents (15–18 years old) and two were household surveys of adults (18–70 years old). Although the studies used different instruments and questionnaires, the results can be broadly summarized as follows. In three studies of school children, prevalence of any offending (measured using 6 or more questionnaire items) was 39% (Guadalupe, 2007), 45% (Assis, Avanci, Santos, Malaquias, & Oliveira, 2004) and 69% (Kahn et al., 1999). In four studies of school children and one study of adolescents in the community, the prevalence of involvement in fights ranged from 15.2% to 22.8% (Abramovay, 2012; Baggio, Palazzo, & Aerts, 2009; Carlini-Cotrim, Gazal-Carvalho, & Gouveia, 2000: samples A&B; da Silva et al., 2009). Prevalence rates of school children carrying firearms were: 1% (Abramovay, 2012), 2.8% (Carlini-Cotrim et al., 2000: sample B) and 4.8% (Carlini-Cotrim et al., 2000: sample A). Among adolescents in households, 9.6% reported carrying any weapon in the previous year (da Silva et al., 2009). The only study examining theft reported a 5% prevalence of stealing at school in the previous year (Abramovay, 2012). In two household surveys of adults (18–70 year olds), the prevalence of hitting a non-family member in the previous year was 5.6% and 2.5% (Orpinas, 1999: samples A and B).

#### 4.3.4 Drug use in national household surveys

Instead of systematically reviewing small-scale studies of drug use in Brazil, we summarize results from several nationwide surveys. The most recent national survey of adult drug use (in 2005) included 7939 respondents in 108 cities with over 200,000 inhabitants, and applied a questionnaire originally developed by the U.S. Substance Abuse and Mental Health Services Administration (Carlini et al., 2006). The prevalence of lifetime use of any drug (excluding tobacco, alcohol and drugs for medical use) among Brazilian adults was 22.8% in 2005, compared to 19.4% in a similar survey in 2001, and compared to 45.4% in the United States in 2004. The prevalence of lifetime use of marijuana in Brazil (8.8%) was slightly higher than in Colombia (5.4%), but much lower than in the United States (40.2%) and in the United Kingdom (30.8%). The prevalence of lifetime use of cocaine was 2.9% (Carlini et al., 2006), similar to the United Kingdom (3.0%), higher than in Colombia (1.6%), but well below the United States (11.2%) (Galduróz, Noto, Nappo, & Carlini, 2005). Lifetime use of crack was reported by 0.7% of Brazilians (Carlini et al., 2006). Lifetime abuse of solvents was 6.1% in Brazil, compared to 1.4% in Colombia and 9.5% in the United States (Carlini et al., 2006). Only 0.1% (7 people) responded that they had used heroin in their lifetime, compared to 1.2% in the USA and 1.5% in Colombia.

Galduróz, Noto, Nappo, and Carlini (2004) examined trends in drug use between 1987, 1989, 1993 and 2007, among 10–18 years old students in Brazil's 10 largest state capital cities (average N = 15,000 in each survey). They concluded that there were significant increases in lifetime use, frequent use and heavy use of many drugs among Brazilian youth. Lifetime use of marijuana increased from 2.8% in 1987 to 7.6% in 1997, and cocaine use increased from 0.5% to 2.0%. In another recent national survey of 761 adolescents (14 to 19 years old) 2.8% reported use of any illicit drug in the previous 12 months (Madruga et al., 2012).

In a national study of 2807 street children in Brazil in 2003, prevalence of lifetime drug use was much higher than in household and student surveys: solvents 44.4%, marijuana 40.4%, cocaine and derivatives 24.5% (Noto et al., 2004).

### 4.4 Risk factors for criminal offending

#### 4.4.1 Characteristics of offenders

Of police-recorded offenders in Brazil in 2009, 87% were male^9^; however, other information on offender characteristics is lacking in national police records. Prison statistics provide more detailed information about characteristics of incarcerated offenders in Brazil. In 2011, 471,000 adults (aged 18 and over) were held in Brazil's prisons, 94% of whom were male (Ministerio de Justiça, 2012). Most prisoners were young: 55% were under 30 years old. Thirty seven percent of prisoners were white, 17% were black, 44% mixed race and 2% other race. Most prisoners had low educational qualifications: 63% had completed less than 10 years of education (*educação fundamental*). Of the 494,000 crimes for which prisoners were incarcerated, 15% were homicides (including theft following homicide: *latrocinio*), 27% were robberies, 18% were other types of property crimes (primarily theft), 25% drugs crimes, 4% sex crimes, 6% weapons crimes, 1% kidnappings, and 35% other crimes.

#### 4.4.2 Risk factors for crime

In a systematic review of the literature, we found nine community based studies that examined individual, family or community risk factors for criminal behavior in Brazil. We summarize the results from these studies that are significant at the *p* < .05 level (unless stated otherwise). We report strength of associations between risk factors and offending in terms of odds ratios wherever possible. The odds of criminal behavior is equal to the number of offenders divided by the number of non-offenders. The odds ratio (OR) equals the odds among people who have a risk factor divided by the odds for people without the risk factor. The OR thus represents how more or less likely people with a risk factor are to commit crime compared to people without the risk factor. An OR larger than 1.0 shows an increased probability of risk whereas an OR smaller than 1.0 shows a reduced probability of risk; an OR of 2.0 or larger indicates strong prediction (Cohen, 1996). If studies did not report odds ratios, we calculated them based on 2 × 2 tables wherever possible.

Assis et al. (2004) (see Table 2) examined rates of offending according to whether or not school students (11–19 years old) had experienced maltreatment (severe physical aggression by parent/carer, psychological maltreatment by significant other, or sexual maltreatment by parents) in São Gonçalo (RJ). Among those maltreated, 58% self-reported offending compared to 30% among the control group (equivalent to a significant odds ratio of 3.2).

Avanci, Assis, Oliveira, Ferreira, and Pesce (2007) examined the association between mental health problems and delinquency in a sample of 1923 students (aged 10–19 years old) recruited in public and private schools in São Gonçalo (RJ). The association was significant in univariate analysis (no further information reported), but not significant in multivariate models that accounted for additional individual, family and school risk factors.

Caicedo, Gonçalves, González, and Victora (2010) examined early bio-social risk factors (measured between birth and age 4) for conviction for violence between ages 12–25, in a prospective birth cohort study of 5228 children born in Pelotas (RS) in 1982. Males were more likely (3.0%) than females (1.0%) to be convicted for violence at least once (OR = 3.1). For both males and females, conviction was associated with maternal black or mixed skin color (males OR = 1.8, females OR = 2.4) and lower family income, in both bivariate and multivariate models. Being in the lowest family income group carried about 10 times the risk of violent conviction compared with being in the highest income group. For males only, a higher number of younger siblings also predicted violent conviction. For females only, having a teenage mother was predictive (OR = 3.9). The following variables were not significantly predictive for either sex: maternal smoking in pregnancy, obstetric complications, low birthweight, duration of breastfeeding, mother's marital status, and number of older siblings.

Carlini-Cotrim et al. (2000) (see Table 2) investigated rates of weapon carrying and fighting among 871 12–18 year-old students in public schools and 804 students in private schools with high fees in the city of São Paulo (SP). Males were more likely than females to be involved in fights (OR = 2.9 public schools; 4.4 private schools) and to carry guns (OR = 7.2 public schools; OR = 27.9 private schools). In public schools, older students (15–18 years old) were more likely to carry guns than younger students (12–14 years old; OR = 2.1), but there was no difference in their rates of fighting. In private schools, older students were less likely to be involved in fights than younger students (OR = 0.7), but there was no significant difference in gun carrying.

In da Silva et al.'s (2009) (see Table 2) study of 960 adolescents (15–19 years old) in Pelotas (RS), males were more likely than females to fight (32.7% versus 13.7%; OR = 4.6) and to carry a weapon (15.8% versus 3.9%; OR = 3.0). Fighting and carrying a weapon were also associated with: working (OR fight = 1.6; OR weapon = 2.8), not practicing religion (OR fight = 1.6; OR weapon = 1.7), using drugs (OR fight = 3.3; OR weapon = 3.3), drinking alcohol (OR fight = 2.9; OR weapon = 3.0), and smoking tobacco (OR fight = 1.9; OR weapon = 2.5). Fighting (but not weapon carrying) was associated with mental health problems (OR = 1.5) and not currently studying (OR = 1.8). Variables not significantly associated (in bivariate tests) with either fighting or carrying weapons were: age, socioeconomic position, and not living with both parents.^10^ In multivariate models, fighting was associated with male sex, use of drugs, drinking alcohol, and mental health problems. In separate multivariate models, weapon carrying was associated with male sex, low socioeconomic class, not studying, working, drug use, drinking alcohol, and mental health problems.

In Guadalupe's (2007) (see Table 2) study of 3637 school children (7–18 years old) in the Belo Horizonte metropolitan area (MG), predictors of self-reported delinquency were assessed in a multivariate logistic regression model. Delinquency was associated with male sex (OR = 3.6), lacking religious belief (OR = 1.6), lower age, lower school attachment, earlier expected age for leaving school, school friendships, lower family wealth, family or friends arrested, and victimization. Delinquency was not significantly associated with race, involvement in school activities, school discipline, physical disorder of the school area, social disorder in the school, or type of school.

In Kahn et al.'s (1999) (see Table 2) study of 710 school students (about 13–19 years old) in São Paulo (SP), boys were significantly more likely than girls to participate in each of 10 (out of 11) different types of delinquent acts. Smoking cigarettes, drinking alcohol, and using marijuana were all significantly associated with students committing more delinquent acts.

In Orpinas' (1999) (see Table 2, Sample A) household survey of 1384 adults in Salvador (BA), men were significantly more likely than women to have hit a non-family member (OR = 1.9). There was no significant difference between men and women among 1114 adults in Rio de Janeiro (Orpinas, 1999; see Table 2, Sample B).

Pacheco (2004) conducted a case–control study of familial risk factors for juvenile delinquency in the city of Porto Alegre (RS). She compared characteristics of 148 male adolescents in juvenile justice institutions with 163 controls recruited from nine schools in the same city. Offenders were less likely than non-offenders to live with their mother (71% versus 87%; OR = 0.4) or father (37% versus 57%; OR = 0.4). Offenders had more siblings on average (mean = 4.3) than non-offenders (mean = 2.6). Offenders were more likely to have used drugs (87% versus 31%, OR = 14.9). They were also more likely to report that family members used alcohol (63% versus 46%; OR = 2.0) and drugs (42% versus 15%; OR = 4.1). Offenders were also more likely to report that family members were involved in crime (55% versus 22%; OR = 4.3) and had relationships characterized by conflict (45% versus 29%; OR = 2.0). Rates of authoritarian, authoritative, indulgent and negligent parenting were also compared between offenders and non-offenders, but there were no significant differences on these variables.

## 5 Discussion

### 5.1 Summary of findings

Key findings from this review on crime and violence in Brazil are as follows. There has been an enormous rise in homicides in Brazil over the last three decades. Brazil has the highest years of life lost to violence out of any WHO member state. Victims of homicide in Brazil are most likely to be young, male, and black. Time trends in non-lethal criminal victimization are complex: hospital admissions data suggest a decline in serious non-lethal assault over the last two decades, but reported physical aggression increased between 1988 and 2009, as did robbery and theft. Brazil appears to have similar levels of overall criminal victimization as several other Latin American and North American countries, but higher rates than Spain. The number of incarcerated juveniles and adults in Brazil has grown substantially in the last two decades. However, Brazilian criminal justice data do not provide for a detailed analysis of the flow of offenders through the system. Self-reported offending studies are in their infancy in Brazil, but indicate quite high levels of offending among school students. National surveys suggest lower rates of drug use in Brazil compared to other countries such as the United States, but there is evidence of significant increases in drug use among Brazilian youth in recent years. Individual and family-level risk factors for crime identified in Brazil are similar to those in high-income countries.

### 5.2 Explaining homicide trends in Brazil

Homicide rates in Brazil are historically high: 26.2 per 100,000 people in 2010, compared to an estimated average of 19 per 100,000 in Europe the 16th century (Eisner, 2003), and 7.9 worldwide in 2008. The number of homicides in Brazil between 1997 and 2007 were more than the number of deaths in numerous recent wars and civil wars in other countries (Waiselfisz, 2010, p. 145).

Many possible causes of the high homicide rate in Brazil have been considered in the literature. Imbusch, Misse, and Carrión (2011) discuss the historical context in Latin America, including the cultures of the Mayas, Incas and Aztecs, colonization by the Portuguese and Spanish, slavery, military dictatorships, and transitions to democracy in the context of economic instability in the late 20th century. They suggest that the increase in homicide rates in Brazil since democratization in the 1980s was caused by a combination of increased inequality, disorganized urbanization, availability of firearms, weak social institutions, and drug trafficking, together with cultural characteristics and a democracy that guarantees political but not social rights. Briceno-Leon, Villaveces, and Concha-Eastman (2008) also emphasize the significance of increases in inequality in many Latin America countries during the 1980s, as well as rises in youth unemployment, increasing consumer aspirations, and loss of traditional mechanisms of social control — particularly within the family and religious institutions. Reichenheim et al. (2011) additionally cite issues of land conflicts, turf wars between gangs, police violence, and an unprecedented growth in the youth population following the baby boom in the 1960s (see also Peres et al., 2011).

Probably the most comprehensive empirical analysis of homicide trends in Brazil was conducted by Cerqueira (2010). He developed seven national indicators of socioeconomic changes between 1980 and 2007 and related them to homicide trends through those years. The seven indicators were: national income, inequality levels (Gini index), the proportion of young males (15–24 years old) in the population, firearm availability, use of illegal drugs, police numbers, and rates of incarceration. Changes in these variables explained about two thirds of the variation in homicide rates between 1980 and 2007. The most important factors explaining homicide increases in the 1980s were the growth in inequality, the increased availability of weapons, and the rise in drug use. During the 1990s, in which incomes generally rose in Brazil, increases in homicide rates were not explained by socioeconomic factors, but rather by the increased proportion of young men in the population (many of whom were unemployed) and the proliferation of firearms (coinciding with a massive growth in the private security industry). Between 2001 and 2007, homicide rates in Brazil declined in line with reductions in inequality, increases in income, a smaller proportion of young-males in the population, and increases in incarceration rates.

In addition, in 2003, national legislation in Brazil criminalized ownership of unregistered guns and carrying of guns on the street, increased prison time for violations of firearm laws, and further restricted the importation of firearms. The downturn in homicide rates after 2003 provoked some optimism about the effects of this legislation (Cerqueira, 2010; de Souza, Macinko, Alencar, Malta, & Neto, 2007). However, the overall reduction in firearm mortality in Brazil between 2003 and 2004 mainly reflected a very sharp drop in homicides in São Paulo state, which accounts for about 25% of national firearm deaths (Goertzel & Kahn, 2009). In other states, rates of firearm death actually increased from 2003 to 2004, leading Goertzel and Kahn (2009, p. 405) to suggest that “The most important factor does not seem to be the passing of national [disarmament] legislation but the vigor with which the legislation is enforced at the state level”.

### 5.3 Patterns of non-lethal crime and violence

According to two large cross-national surveys, overall levels of criminal victimization in Brazil are similar to other countries in the Americas. Thus, the characterization of the United States as having a homicide problem not a crime problem (Zimring & Hawkins, 1999) might also apply Brazil — the important difference being that homicide levels in Brazil are even higher than in the United States (see Table 1). However, specific types of non-lethal crime (e.g., vehicle theft and robbery) may be more common in Brazil than elsewhere, and some types of crime may be less common (e.g., physical assault). This was suggested by one victimization study in four large Brazilian cities. However, without more detailed, national-level victimization data that are comparable with other countries, it is not clear whether Brazil's crime problem is limited to homicide or whether other types of crime are also higher than in other countries.

The rate of hospitalization for non-lethal violence in Brazil declined from the mid-1980s through 2010. One possible explanation for this trend is that, as firearm availability increased and homicide rates went up, fewer victims survived serious assaults. Consistent with this hypothesis, national survey evidence suggests that overall physical aggression increased between 1988 and 2009. More detailed survey information on trends in different types of assault (less and more serious assault) would be required to confirm whether serious *non-lethal* violence declined only because more victims died from assault.

Brazil has not participated in comparative research such as the International Self-Report Delinquency Study (Junger-Tas et al., 2010) making it hard to compare levels of self-reported offending in Brazil with other countries. However, rates of general offending by school children appear high in Brazilian studies: ranging between 39% and 69% in three surveys. The following risk factors for offending were replicated in at least two Brazilian studies in this review: male sex, cigarette use, alcohol use, drug use, mental health problems, lacking religious belief/practice, low family income, large family (many siblings), and family or friends involved in crime. All of these risk factors have been found in numerous studies in high-income countries (Ellis et al., 2009; Murray & Farrington, 2010; Rutter, Giller, & Hagell, 1998). Therefore, the evidence to date does not suggest that individual or family level risk factors are different in Brazil compared to high-income countries (unlike some community factors: Villarreal & Silva, 2006). Nonetheless, the range of risk factors that have been examined in Brazil is limited, and new larger studies encompassing other risk factors might reveal differences not identified to date. In a systematic review about the prevalence of and risk factors for childhood conduct disorder in Brazil, a similar conclusion was reached about future research needs (Murray et al., 2013).

A critical issue for research is identifying which risk factors actually cause increases in crime, as opposed to merely mark genetic transmission or other environmental risk mechanisms (Jaffee, Strait, & Odgers, 2012; Rutter, 2003). Several Brazilian studies used regression models to statistically control for confounding factors, but we found no research using other methods to investigate causal risk effects. There is a need for new research to establish which risk factors are actually causes of crime in Brazil, including studies with genetically sensitive designs (e.g., twin studies), natural experiments, propensity score matching, and analyses of within individual change over time (Jaffee et al., 2012; Murray, Farrington, & Eisner, 2009; Rutter, 2003). Risk mechanisms should also be investigated in Brazil that have not been extensively studied in high-income countries, for example effects of malnutrition. In a prospective cohort study in Mauritius, malnutrition at age three years was predictive of aggression up to age 18 independently of psychosocial adversity (Liu, Raine, Venables, & Mednick, 2004). No similar Brazilian studies were found, and future research on crime in Brazil should include such risk factors, which are more common there than in high-income countries.

### 5.4 Limitations

The available data in Brazil and the number of primary studies of course limited our review. Although vital statistics on homicide are perhaps a reasonably trustworthy source of information, there is no doubt that Brazilian police data are not very reliable or complete. Additionally, the fact that Brazil has not participated in major international programs of research on victimization or self-report delinquency means that conclusive evidence is lacking on how rates of non-lethal crime and violence compare between Brazil and other countries. Within the studies that we did assess, heterogeneous samples and measures meant that we could not conduct a meta-analysis of their results. Existing studies focused almost exclusively on psychosocial risk factors, and we were unable to consider biological mechanisms that may interact with environmental factors to cause crime and violence in Brazil.

### 5.5 Future research needs

A key conclusion of this review is that there is great need for more systematic data collection on crime and violence in Brazil. In particular, police recording of offenses and offenders needs to be conducted systematically across the country, and records compiled nationally to monitor numbers of officially recorded crimes and offenders, and characteristics of offenders in Brazil. Progress is being made to monitor injuries presented at Brazilian public hospital emergency departments (Malta et al., 2012). If all hospitals participated in such a system, this would provide invaluable additional information on violence in Brazil, and the resulting data could be used in collaboration with police as part of violence prevention programs (Shepherd, 2007).

There is also an urgent need to mount regular national victimization surveys in Brazil to provide reliable estimates of crime frequency and time trends that can be compared with official statistics and with other countries. In 2010, Brazil conducted its first national victimization survey using the standardized instrument from the International Crime Victimization Survey (results still unavailable). Given that reducing violence is one of the main challenges facing Brazil today, it is imperative to continue collecting this basic information to monitor progress made. Improved victimization data should also be supplemented by large-scale research on self-reported delinquency, ideally as part of the International Self-Reported Delinquency Studies. Longitudinal studies should be conducted to examine risk factors for crime from childhood onwards. Large-scale birth cohort studies focusing on public health outcomes exist in several LMIC, including several in Brazil (Batty, Alves, Correia, & Lawlor, 2007). Although public health problems have been and continue to be major challenges, the enormous costs of crime and violence also necessitate investment in longitudinal research on these topics in Brazil and other LMIC.

## Figures and Tables

**Table 1:** Worldwide homicide rates in 2008 and Disability Adjusted Life Years (DALY) caused by violence in 2004.

		Homicides (per 100,000)	DALY (thousands)
World		7.9	21,701,428
High income countries		2.7	886,297
Low–middle income countries	Africa	20.1	6,333,294
	The Americas	24.1	6,024,751
	Eastern Mediterranean	3.9	1,346,008
	Europe	9.8	1,826,177
	South-East Asia	5.8	3,444,677
	Western Pacific	2.8	1,775,947
Guatemala (1)		61.3	175
El Salvador (2)		54.9	113
Côte d'Ivoire (3)		52.5	324
Brazil (13)		29.6	2488
South Africa (17)		27.3	1006
Russian Federation (39)		18.4	1276
Mexico (43)		17.9	415
Indonesia (76)		8.3	611
United States of America (93)		6.2	599
India (102)		4.4	1866
Pakistan (108)		3.4	174
Turkey (113)		2.8	67
China (142)		1.6	993
Iran (150)		1.4	89
France (152)		1.4	17
United Kingdom (162)		1.1	33
Italy (163)		1.1	15
Germany (178)		0.8	19
Egypt (185)		0.6	27
Japan (189)		0.5	21

Source. Adapted from World Health Organization Global Burden of Disease tables (http://www.who.int/healthinfo/global_burden_disease/, accessed 9/3/2012). Table shows selected WHO member states with homicide rate rank in parentheses (out of 193 countries).

**Table 2:** Self-report offending studies with prevalence estimates in Brazil.

Study	Location	Sample	Number of participants (% response)	Age range	% Male	Questionnaire items	Reference period	Outcome	Prevalence
Abramovay (2012) See also Abramovay and Rua (2006)	Urban: Belém, PA; Porto Alegre, RS; Salvador, BA; São Paulo, SP; Distrito Federal	Students in random selection of classes in random selection of 113 public schools	10,069 (~ 75%)	~ 6–17	~ 45%	3 questions on: theft at school, fights (hit someone at school), carried weapons into school	1 year 1 year Lifetime	Any theft Fights Carried firearm	5% 16% 1%
Assis et al. (2004)	Urban: São Gonçalo, RJ	Students in random selection of 44 classes in 38 schools	1685 (100%)	11–19	56%	7 questions on: falsification of documents, destruction of property, bullying, fighting, weapon carrying, theft/robbery	Not reported	Any offending	45%
Baggio et al. (2009)	Urban: Porto Alegre, RS	Students in random selection of school classes	1170 (86%)	12–18	47%	Involvement in fights	Not reported	Fights	19%
Carlini-Cotrim et al. (2000) Sample A	Urban: São Paulo, SP	Students in 4 classes in random selection of 10 public schools	871 (72%)	12–18	47%	Involvement in fights (twice or more); carrying a firearm	12 months 12 months	Fights Carried firearm	15.2% 4.8%
Carlini-Cotrim et al. (2000) Sample B	Urban: São Paulo, SP	Students in 7 private schools with high monthly fees	804 (90% in participating schools: 7 out of 9)	12–18	49%	Involvement in fights (twice or more); carrying a firearm	12 months 12 months	Fights Carried firearm	20.3% 2.8%
da Silva et al. (2009)	Urban: Pelotas, RS	All adolescents in 86 households in 90 randomly selected census districts	960 (92%)	15–18	48%	Involvement in fights; carrying weapons	12 months 12 months	Fights Carried weapon	22.8% 9.6%
Guadalupe (2007)	Urban: Belo Horizonte, Contagem, Betim, Ibirité, Ribeirão das Neves, Santa Luzia, MG	Random sample of students in 65 public and private schools	3637 (N.R.)	7–18	N.R.	6 questions on: taking weapons to school, fights in school, theft in school, robbery, gang membership	Lifetime to current	Any offending	39%
Kahn et al. (1999)	Urban: São Paulo, SPP	Students in 4 participating public schools and 3 private schools	710 (67% in participating schools: 7 out of 40)	~ 13–19	53%	11 questions on delinquency in school: falsification of documents, destruction of property, fights, aggression, harassment, theft, weapon carrying	Lifetime	Any offending	69.4%
Orpinas (1999) Sample A	Urban: Salvador, BA	Adults in clustered samples of households	1384 (N.R.)	18–70	46%	3 questions on aggression against non-family member	12 months	Hit non-family member	5.6%
Orpinas (1999) Sample B	Urban: Rio de Janeiro, RJ	Adults in clustered samples of households	1114 (N.R.)	18–70	43%	3 questions on aggression against non-family member	12 months	Hit non-family member	2.5%

N.R. = Not reported.

## Footnotes

1 Homicide and DALY data from the World Health Organization were downloaded from http://www.who.int/topics/global_burden_of_disease/en/ on 9 March 2012 and might include updated information compared to the report Global Burden of Disease 2004 Update (World Health Organization, 2008).

2 Either civil or military or both types of police data available in Acre, Amazonas, Bahia, Distrito Federal, Espírito Santo, Goiás, Mato Grosso, Mato Grosso do Sul, Paraíba, Piauí, Rio de Janeiro, Rio Grande do Norte, Rondônia, Santa Catarina, São Paulo, Sergipe, and Tocantins.

3 Because coding changed from ICD9 to ICD10 in 1998, data prior to 1998 are standardized to the 1997 rate. Data after 1998 are standardized to the 1998 rate.

4 Also, the proportion of the population with private health insurance was 25% in 1998, 25% in 2003 and 26% in 2008 (Instituto Brasileiro de Geografia e Estatística, 2010a).

5 In states classified as having the best quality police data rates were slightly higher: 21 attempted homicides, 567 robberies, 421 assaults, 16 rapes, 46 crimes of drug possession, 48 crimes of drug trafficking, and 20 crimes of firearm possession. These states include Distrito Federal, Goiás, Maranhão, Mato Grosso, Mato Grosso do Sul, Paraíba, Piauí, Rio Grande do Sul, Rondônia, Roraima, Santa Catarina, São Paulo, Sergipe, Tocantins, with total population of 90.8 million. Ceara and Paraná were excluded because they returned too little information on specific crime types.

6 We do not examine data from previous years which had different sampling frames: between 31 and 33% of the national population in 1995–1997 and 11.6% of urban residents in 1998–2000.

7 For vehicle related crime (theft of car, theft of motorbike, theft of bike, theft from car) results pertain only to respondents who owned relevant vehicles.

8 Distrito Federal, Goiás, Mato Grosso, Mato Gross do Sul, Paraíba, Piauí, Rondônia, Roraima, Sergipe (total population = 24.8 million).

9 Among 93% of cases for whom sex was recorded.

10 In separate analyses of the same study, Horta, Horta, Pinheiro, and Krindges (2010) showed that living with *neither* biological parent was associated with increased risk of weapon carrying.
